# The Book World of Medicine and Science

**Published:** 1899-11-11

**Authors:** 


### THE BOOK WORLD OF MEDICINE AND SCIENCE.

Faith-Healing and "Christian Science." By Alice Feilding. (London: Duckworth and Co. 1899. Price 3s. 6d. net.)

It may perhaps appear to some that the author of this book takes the subject of Christian Science somewhat too seriously, and pays to its professors an undue compliment in treating its vagaries in the careful manner in which she deals with its grotesque claims. But when one bears in mind to what an extent this fanciful sect has spread among a portion of society, which, to say the least of it, ought to know better than to allow itself to be led by the nose by trickery and a clap-trap title, it is perhaps as well, in considering its teachings and its tendencies, not to trust entirely to the kind of ridicule which naturally rises to the lips of educated men at the very mention of its preposterous creed. In a very interesting and suggestive chapter the author shows how ancient and universal has been the practice of treating certain diseased conditions by the excitation in the minds of the sufferers of a feeling of expectancy which may best be described as faith. This "healing of the body by mental agency" is, she says, a phenomenon which, generally under the direct auspices of religion, whether Pagan or Christian, has manifested itself from time to time from the earliest days of history, and she gives a long series of examples in which cures have been supposed to follow a large variety of proceedings, which seem to have had only this in common, that they raised a condition of expectancy and of faith. The records of all people, savage and civilised, teem with examples of what have been regarded as supernatural or miraculous cures, and perhaps the best part of the book before us is the demonstration that whatever of the strange and the unexplained may be brought forward by any modern sect as proof of the miraculous powers of their priesthood, these things are but of the same nature as, and often, indeed, are but very poor examples of that influence of the mind upon the body which is well recognised as having been at the root of "miraculous" cures in many ages. In regard to the individuals who have appeared to possess the psycho-therapeutic power to become faith healers, we are told that they "may be divided into two classes, thus: (1) Those who have practised it in a truly devout, simple, and single-minded spirit, who laboured for love of God and His creatures and not for gain. (2) Those, and perhaps the largest number, who practised for greed of what Timothy so justly described as 'filthy lucre,'" and it is very properly added that the distinction between these two classes is not always easy to draw. It is hard, if not impossible, to distinguish the true motive power, for, even granting honesty of purpose, self-deception is a most common failing, and "when the deception is one favourable to personal pretensions, is it surprising that it should take root the more easily?" Coming now to Christian Science, it is pointed out that the foundations of this form of religion, for so we suppose it must be called, "rest on what is technically called 'thaumaturgy,' the art of working wonders and miracles," and in view of what has been so well demonstrated in the preceding chapter it is shown that at the very most, accepting many of their statements as being the result of honest conviction, the cures claimed by Christian Science are but fresh examples of the endless varieties of faith-healing exercised by other cults and religious systems, such as the Salvation Army faith healers, the New Forest Shakers, the Mormons, the Peculiar People, &c., all only differing from each other, and from Lourdes, in their several systems of doctrinal teaching. We think that the author of this book is perhaps somewhat over liberal-minded, and that she is too ready to admit honest motives in people whose surroundings are such as to suggest much dishonesty; yet after all her careful attempt to find out the truth of the matter in regard to these Christian Scientists, this is what she says: "At any rate, this creed, invented, demonstrated, expounded, and diffused by American ladies, is surely the natural outcome, in emotional natures and untrained minds, of a smattering of spiritualism, mesmerism, mental therapeutics, mysticism, and metaphysics, coupled with a profound and lofty disdain for the most elementary scientific knowledge." Finally we may quote, as she has done, the following passage from an article by Dr. A. T. Myers and Mr. F. W. H. Myers, in the proceedings of the Society for Psychical Research: "The existence of a crew of this kind is an inevitable, although it may be hoped transitory, result of any wide-spread popular attempt at cure by suggestion and self-suggestion." The whole book is more or less interesting, but we do not think that the portion devoted to Christian Science is the best part of it.

Stone, Prostate, and Other Urinary Disorders. By Reginald Harrison, F.R.C.S., &c. (London: J. and A. Churchill. 1899. Pp. 190. Price 5s.)

In this volume Mr. Harrison has given us, from his many writings, a selection of essays which exemplify admirably his ripe experience and mature judgment. Naturally these papers deal (but in no spirit of narrow specialism) with that department of surgery with which Mr. Harrison's name is peculiarly identified. Most, if not all of them have previously been published, but all deserve more than the passing attention which too often is all that is afforded to reports of clinical lectures. From each of these dozen or more papers something can be learnt, some hints gathered, some warning stored up. But we are especially instructed by the papers on vasectomy and the treatment of albuminuria by reni-puncture. Mr. Harrison, while eager to afford his patients all the benefits that may be derived from the newer procedures, exhibits a careful conservatism that has enriched surgery by such a valuable alternative to more drastic methods, as section of the vas deferens; a method of use in more than one morbid condition. Mr. Harrison's acute surmises and remarkable experiences in the matter of reni-puncture are almost, if anything, of greater interest to the physiologist and physician than to the surgeon. Nevertheless, it is likely that Mr. Harrison has found a clue to the puzzling problem of success following apparently fruitless exploratory operations, and has suggested a method of relief that must not escape consideration in certain cases. Mr. Harrison's papers are admirably and incisively written, and are a happy example of the value, not so much of specialism as of the attention of a surgeon of wide experience to special problems, and of a felicitous combination of enthusiasm and caution.

